# Convolutional neural network classification of ultrasound parametric images based on echo-envelope statistics for the quantitative diagnosis of liver steatosis

**DOI:** 10.1007/s10396-024-01509-w

**Published:** 2024-11-23

**Authors:** Akiho Isshiki, Kisako Fujiwara, Takayuki Kondo, Kenji Yoshida, Tadashi Yamaguchi, Shinnosuke Hirata

**Affiliations:** 1 Department of Medical Engineering, Graduate School of Science and Engineering, Chiba University, Chiba, 263-8522 Japan; 2 Department of Gastroenterology, Graduate School of Medicine, Chiba University, Chiba, 260-8677 Japan; 3 Ultrasound Center, Chiba University Hospital, Chiba, 260-8677 Japan; 4 Center for Frontier Medical Engineering, Chiba University, 1-33 Yayoicho, Inage-ku, Chiba-shi, Chiba 263-8522 Japan

**Keywords:** Liver steatosis, Ultrasound attenuation, Echo-envelope statistics, Convolutional neural network

## Abstract

**Purpose:**

Early detection and quantitative evaluation of liver steatosis are crucial. Therefore, this study investigated a method for classifying ultrasound images to fatty liver grades based on echo-envelope statistics (ES) and convolutional neural network (CNN) analyses.

**Methods:**

Three fatty liver grades, i.e., normal, mild, and moderate-to-severe, were defined using the thresholds of the magnetic resonance imaging-derived proton density fat fraction (MRI-PDFF). There were 10 cases of each grade, totaling 30 cases. To visualize the texture information affected by the deposition of fat droplets within the liver, the maps of first- and fourth-order moments and the heat maps formed from both moments were employed as parametric images derived from the ES. Several dozen to hundreds of regions of interest (ROIs) were extracted from the liver region in each parametric image. A total of 7680 ROIs were utilized for the transfer learning of a pretrained VGG-16 and classified using the transfer-learned VGG-16.

**Results:**

The classification accuracies of the ROIs in all types of the parametric images were approximately 46%. The fatty liver grade for each case was determined by hard voting on the classified ROIs within the case. In the case of the fourth-order moment maps, the classification accuracy of the cases through hard voting mostly increased to approximately 63%.

**Conclusions:**

The formation of parametric images derived from the ES and the CNN classification of the parametric images were proposed for the quantitative diagnosis of liver steatosis. In more than 60% of the cases, the fatty liver grade could be estimated solely using ultrasound images.

## Introduction

In recent years, the number of patients with metabolic dysfunction-associated fatty liver disease (MAFLD) has rapidly increased and is estimated to be approximately 25% of the total population [1, 2]. MAFLD is strongly associated with the development of liver fibrosis, which is a major contributor to cirrhosis and hepatocellular carcinoma [3–5]. Hence, early detection and quantitative diagnosis of liver steatosis and fibrosis are crucial.

Hepatic fat deposition can be estimated using the magnetic resonance imaging-derived proton density fat fraction (MRI-PDFF) [6–8]. MRI-PDFF is a reliable diagnostic modality, but it is costly and time-consuming. Moreover, the number of medical facilities capable of performing MRI-PDFF is currently limited. Ultrasonography is an inexpensive and time-saving procedure. The progression of hepatic fat deposition increases the attenuation of the ultrasound signal [9]. Equipment and applications in ultrasound scanners, such as FibroScan (controlled attenuation parameter: CAP) and attenuation imaging (ATI), have been implemented to estimate the attenuation of the ultrasound signal or image [10–14]. However, the estimated ultrasound attenuation also includes components from scatterers and tissue structures. This reduces the accuracy of the estimations from ultrasound images. The accuracy of diagnosing liver steatosis using the CAP has been reported to be inferior to that of MRI-PDFF [11, 15].

The texture of ultrasound images of the liver varies due to the deposition of fat droplets and the replacement with fibrous tissues. The texture information has been quantitatively represented by the statistics and probability density function (PDF) of the pixel brightness, which is echo envelopes, and more advanced methods have also been studied for tissue characterization [16–21]. Therefore, the effects of fat droplets and fibrous tissues can be evaluated via these methods of echo-envelope statistics (ES) analysis. A quantitative diagnosis of the liver can be performed by evaluating the ES and the PDF. However, the results of these analyses do not correspond only to the amount of fat droplets or fibrous tissues but also to the number density and distribution of various types of scatterers. Therefore, the fatty liver grade and liver fibrosis stage referenced in clinical practice cannot be directly evaluated.

In a previous study, a combination of ES analysis and convolutional neural network (CNN) analysis was proposed for the quantitative diagnosis of liver fibrosis [22]. CNNs have been extensively utilized across various medical fields, including applications in ultrasound images for tasks such as segmentation, beamforming, denoising, and tumor diagnosis [23–29]. A CNN was used to link the ultrasound images with ES to liver fibrosis stage in this method. In ultrasound images of livers without steatosis, the potential for quantitative diagnosis of liver fibrosis through the CNN classification was suggested.

In the present study, the formation of parametric images derived from ES of ultrasound images, and the classification of the parametric images into their fatty liver grades using the CNN, are proposed for the quantitative diagnosis of liver steatosis. In this method, the distribution of moments (the moment map) is utilized as the parametric images. The moment, which is the expected value of the exponential envelopes in the surrounding pixels, is one of the ES and is also associated with the shape of the PDF.

The moments can reflect the progression of liver steatosis, but the change in the moments with the progression vary depending on the order of the moment. The aim of the present study was to reveal the effectiveness of the parametric images and a suitable order of moment. Therefore, moment maps of the first- and fourth-order moments were employed as parametric images. In addition, moment heat maps formed from both moment maps were also employed. The accuracies of the CNN classification of each parametric image for fatty liver grades were then evaluated through transfer learning using VGG-16, a CNN model. Moreover, the fatty liver grades classified using the CNN were compared with the liver steatosis indicators CAP and ATI in each case.

## Materials and methods

### Clinical echo data

Clinical data were obtained from Chiba University Hospital (Chiba, Japan). An ultrasound scanner (Aplio a550; Canon Medical Systems, Otawara, Japan) equipped with a convex array probe (PVT-475BT; Canon Medical Systems) was used to acquire the ultrasound images. The center frequency of the transmitted ultrasound was 3 MHz. For image acquisition, the focal length and maximum depth were fixed at 78 mm and 120 mm, respectively.

The background diseases in all the cases included liver steatosis (LS), MAFLD, metabolic dysfunction-associated steatohepatitis (MASH), primary biliary cholangitis (PBC), autoimmune hepatitis (AH), compensated liver cirrhosis (CLC), and others. The hepatic fat deposition and liver stiffness were investigated at Chiba University Hospital. The hepatic fat deposition measured using MRI-PDFF ranged from 1.9 to 23.8%. According to a previous study, the fatty liver grades can be categorized into four groups: grade 0, normal (< 5.2%); grade 1, mild (5.2–11.3%); grade 2, moderate (11.3–17.1%); and grade 3, severe (> 17.1%)[15]. The cut-off area under the receiver operating characteristic (AUROC) values for detecting MRI-PDFF grades ≥ 1, grades ≥ 2, and grade 3 were 0.96, 0.90, and 0.79, respectively. Thus, the classification based on MRI-PDFF is considered reliable. In this study, we defined three fatty liver grades: G0 as normal, G1 as mild, and G2-3 as moderate and severe. This is because the number of moderate and severe cases was insufficient. There were 10 cases of each grade, totaling 30 cases, with four to ten images per case. In the present study, transfer learning of the typical CNNs was performed. Therefore, the number of cases and the number of input images per case were deliberately matched to remove the risk by imbalanced data. The liver stiffness and CAP measured using the FibroScan device ranged from 3.4 kPa to 46.1 kPa and from 142 to 358 dB/m, respectively. The ATI measured using the ultrasound scanner ranged from 0.36 to 1.21 dB/cm/MHz. The image findings related to liver steatosis that can be confirmed in an ultrasound image were verified by physicians. The six image findings are as follows: (1) bright liver, (2) liver-kidney contrast, (3) liver-spleen contrast, deep attenuation, (4) deep attenuation, (5) hepatic and portal vein blurring, and (6) flag sign. Table 1 presents the MRI-PDFF and six image findings of all cases.

**Table 1:** MRI-PDFF and six image findings: (1) bright liver, (2) liver-kidney contrast, (3) liver-spleen contrast, deep attenuation, (4) deep attenuation, (5) hepatic and portal veins blurring, (6) flag sign of all cases

	No.	MRI-PDFF [%]	Imaging findings					
			1	2	3	4	5	6
G0 Normal	1	1.9						
	2	2.3	◯	◯		◯	◯	
	3	2.4						
	4	2.7						
	5	4.2	◯	◯			◯	
	6	4.2	◯					
	7	4.5	◯					
	8	4.6				◯		
	9	4.7	◯	◯	◯		◯	
	10	5	◯			◯	◯	◯
G1 Mild	11	5.4	◯	◯		◯	◯	◯
	12	5.4	◯	◯		◯	◯	
	13	6.2	◯	◯		◯	◯	◯
	14	6.7				◯	◯	◯
	15	7.5	◯			◯	◯	
	16	8	◯	◯		◯	◯	◯
	17	8.4						
	18	8.5	◯	◯	◯	◯	◯	
	19	8.9	◯	◯				
	20	9.4	◯	◯		◯	◯	
G2-3 Moderate and Severe	21	11.9	◯	◯	◯	◯	◯	
	22	12.7	◯	◯		◯	◯	
	23	13.5		◯			◯	
	24	16.6	◯	◯		◯	◯	
	25	18.7	◯	◯		◯	◯	
	26	21.6	◯	◯		◯	◯	
	27	22	◯	◯	◯	◯	◯	
	28	23.8	◯	◯		◯	◯	
	29	25.9	◯	◯	◯	◯	◯	
	30	32.1				◯	◯	

### Normalization of ultrasound images and formation of moment map

Echo envelopes along the scan line of a convex probe with log compression were used. The ultrasound images were reconstructed through the scan conversion of envelopes that were transformed linearly. The lateral and depth pixel spacing of the image was set to 50 μm. Only the liver region was segmented from the images.

**Fig. 1:**
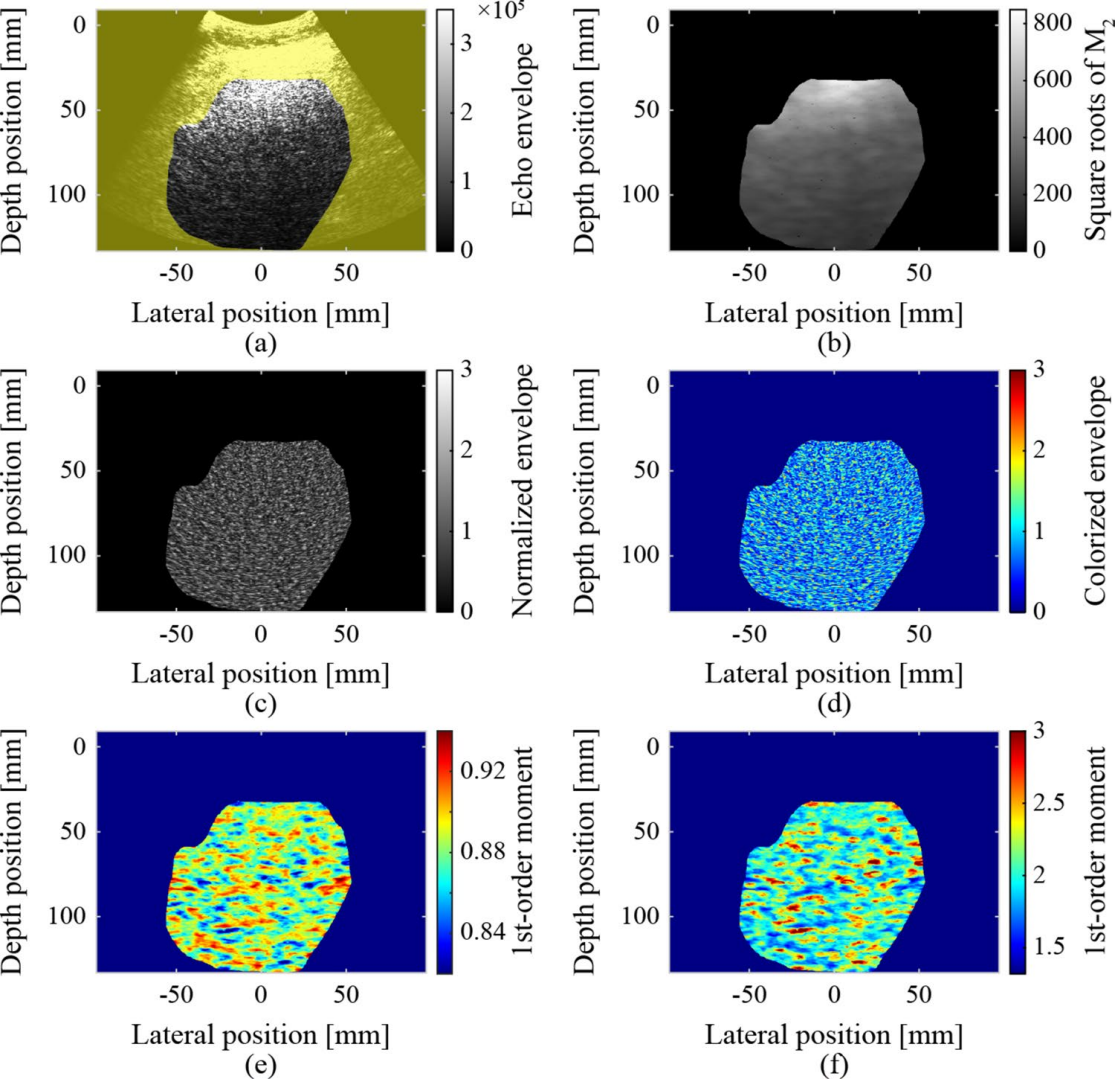
Normalization of ultrasound images and formed moment maps: **a** segmented liver region, **b** square roots of the second-order moments, **c, d** normalized and colorized ultrasound images, **e** first-order moment map, and **f** fourth-order moment map

The ultrasound images were normalized using the square roots of the second-order moments to remove the effects of focus, gain, and attenuation during transmission and reception, as shown in Fig. 1. Simultaneously, non-speckle signals (pixels) that were significantly brighter than the surrounding pixels were removed. The removal threshold was set to three times the square root of the second-order moment. The sizes of the elliptical regions were set to 4.6 mm and 10.8 mm in the depth and lateral directions, respectively, for calculating the second-order moments. The size of this elliptical region corresponds to 6 × 8 times the resolution (0.76 mm × 1.35 mm). The second-order moment in the elliptical region centered on each target pixel was calculated as follows:

$$\:\begin{array}{c}\begin{array}{c}{M}_{n,i,j}=E\left[{x}_{k}^{n}\right],\#\left(1\right)\end{array}\#\end{array}$$

where $\:n$ is the order of the moment, $\:i$ and $\:j$ are the coordinates of the target pixel, and $\:E\left[{x}_{k}\right]$ is the expected value of $\:{x}_{k}$, which is the echo envelope of each pixel in the elliptical region. If the echo envelope of the target pixel exceeded the threshold, the target pixel was excluded as a non-speckle signal. The process of calculating the second-order moments and pixel exclusion was repeated until there were no longer any pixels to exclude. The normalized ultrasound and colorized images inputted to the CNN are also shown Fig. 1. The colorization involved converting from a gray-scale color palette (conventional B-mode style) to a jet color palette. In a previous study, the accuracy of CNN classification of ultrasound image slightly increased as a result of the colorization [22].

The first- and fourth-order moments around each pixel were calculated using Eq. (1) ($\:n=1,\:4$) within an elliptical region centered on the pixel, as shown in Fig. 1. The sizes of the elliptical region were set to 3.0 mm and 8.1 mm in the depth and lateral directions, respectively. The ES can be reliably calculated in regions where the sizes in both the depth and lateral directions are eight times greater than the resolution of the ultrasound image [30]. However, tissue information may be blurred by calculations in such regions. Therefore, the size of the elliptical region used to form the parametric images was reduced to 4 × 6 times the resolution.

### Extraction of region of interest and formation of moment heat map

**Fig. 2:**
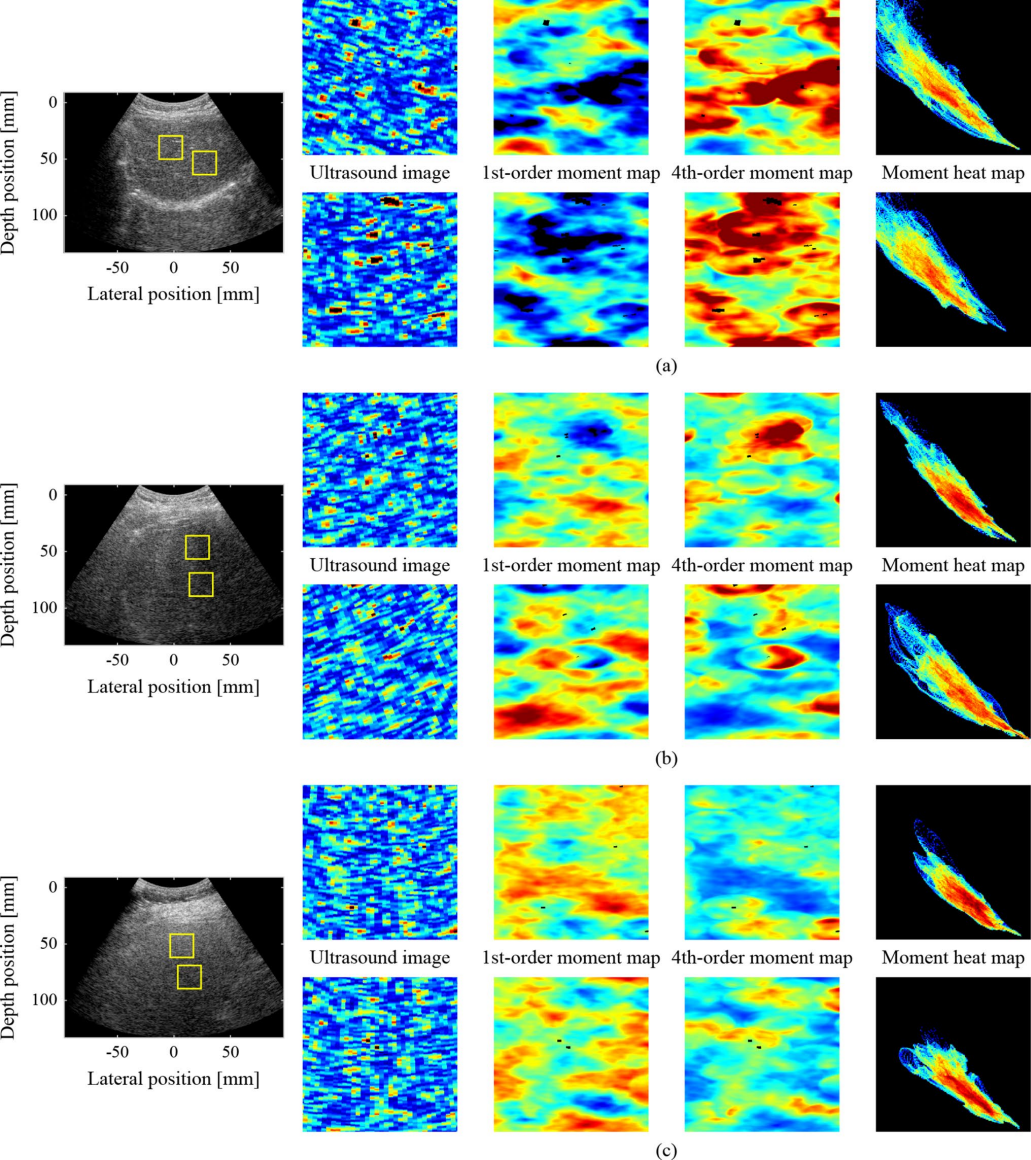
Examples of ROIs as input images at the same locations: **a** case of G0 (No. 4 in Table 1), **b** case of G1 (No. 13 in Table 1), **c** case of G2-3 (No. 26 in Table 1)

The region of interest (ROI) was extracted as an input image for the CNN. The size of the ROI was 20 mm (400 pixels) in the lateral and depth directions. The sliding interval was at least 4 mm. In the extracted ROIs, pixels outside the liver region and pixels excluded by normalization accounted for less than 1%. The ROIs were extracted from the same locations in the colorized ultrasound images, first- and fourth-order moment maps, as shown in Fig. 2.

A moment heat map corresponding to the ROIs in the first- and fourth-order moment maps was also created to show the distribution and relationship of each moment. In the heat map, the 2D coordinates were prepared with the fourth-order moment as the vertical axis and the first-order moment as the horizontal axis. The values of the first- and fourth-order moments for each pixel were then plotted in 2D coordinates. Finally, the number of plotted pixels in each grid was indicated in color. Figure 2 shows examples of the heat maps. The distribution and relationship between the first- and fourth-order moments can be evaluated simultaneously and visually using a heat map.

### Learning and validation of CNN

The ultrasound images, moment maps, and moment heat maps within the ROIs were classified using a CNN. On average, 256 ROIs per case (7680 ROIs) were randomly selected and used for learning, validating, and testing the network. The pretrained VGG-16 in the Deep Learning Toolbox of MATLAB (The MathWorks, Inc., Natick, USA) was employed for the CNN classification of ROIs based on fatty liver grades. For transfer learning, the last fully connected layer of VGG-16 was replaced for the classification of fatty liver grades G0, G1, and G2-3 (input: 4096, output: 3). The weights of the replaced layers were initialized using random numbers.

In transfer learning, only two fully connected layers and two convolutional layers from the output layer were trained using stochastic gradient descent with a mini-batch processing of 64 data points. The dropout rate between the fully connected layers was set at 75%, and the learning rate was 7.5 × 10^−5^$\:.$ For the four-fold cross-validation, 30 cases were divided into four groups, as shown in Table 2. In the four-fold cross-validation, VGG-16 was trained using the ROIs in Groups 1 and 2. Subsequently, the ROIs in Group 3 were classified for validation using the trained VGG-16 at every epoch. Finally, the ROIs in Group 4 were classified for testing using the trained VGG-16 with a minimum validation loss. All the ROIs were classified for testing by rotating the process. The number of epochs was determined based on the validation losses, and it ranged from 7 to 18. In the proposed method, 87 to 425 ROIs were extracted and classified for each case. Therefore, the fatty liver grade for each case was determined through hard voting of the classified ROIs within the case.

**Table 2:** Numbers of utilized cases and ROIs for four-fold cross validation

	Group 1		Group 2		Group 3		Group 4	
	Cases	ROIs (per case)	Cases	ROIs (per case)	Cases	ROIs (per case)	Cases	ROIs (per case)
G0	3	768 (120–450)	2	512 (159,353)	3	768 (232–298)	2	512 (87,425)
G1	3	768 (210–315)	2	512 (184,328)	3	768 (161–344)	2	512 (256)
G2–3	3	768 (182–293)	2	512 (256)	3	768 (107–331)	2	512 (164,348)

## Results

### Evaluation of ultrasound parameters

**Fig. 3:**
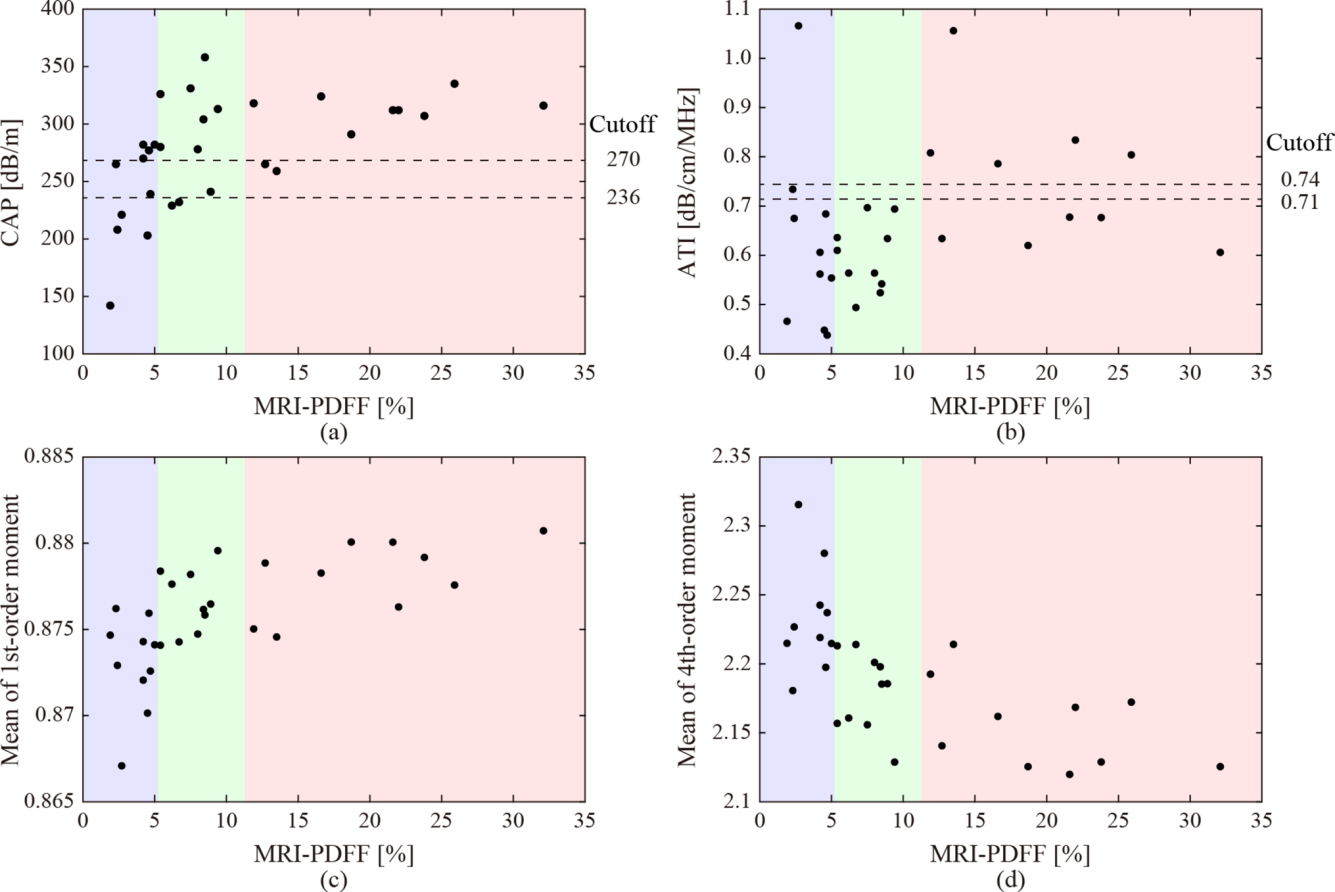
Relationship between hepatic fat deposition (MRI-PDFF) with ultrasound attenuation of **a** CAP, **b** ATI, and ES of the means of **c** first- and **d** fourth-order moments in all the ultrasound images

**Fig. 4:**
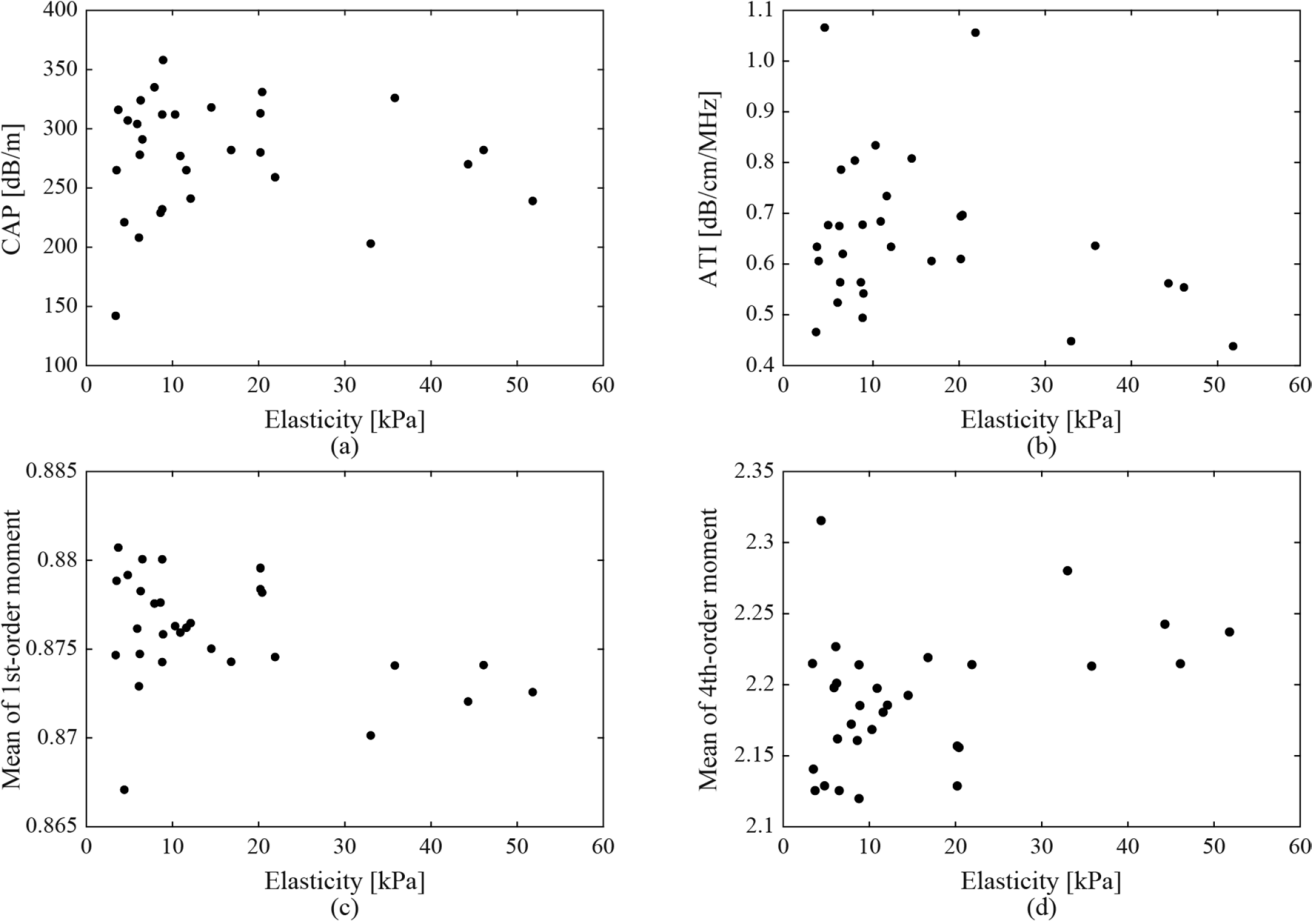
Relationship between liver stiffness (elasticity measured with FibroScan) with ultrasound attenuation of **a** CAP, **b** ATI, and ES of the means of **c** first- and **d** fourth-order moments in all the ultrasound images

Figure 3 shows the relationship between MRI-PDFF with CAP and ATI in each case, the means of the first-order moments, and those of the fourth-order moments in all the ultrasound images in each case. The dashed lines indicate the cut-off values of the CAP and ATI for the fatty liver grades of G0 and G1, G1, and G2-3 [13, 15]. The background colors indicate fatty liver grades by MRI-PDFF. The classification accuracies based on the cut-off values were 43.3% and 20%, respectively. The values of the CAP and ATI, and the means of the first-order moment, were observed to increase in correspondence with the MRI-PDFF. In addition, the means of the fourth-order moment showed a similar decreasing trend. However, the classification of the fatty liver grades using CAP or ATI was not straightforward. Moreover, the impact of liver fibrosis on these ultrasound parameters was investigated. Since liver biopsies were not performed in all cases, liver stiffness (elasticity) measured with FibroScan was used as an indicator. Figure 4 shows the relationship between the elasticities with CAP and ATI in each case, the means of the first-order moments, and those of the fourth-order moments in all the ultrasound images in each case. As for liver stiffness, a significant association was not observed compared to MRI-PDFF.

### CNN classification

**Fig. 5:**
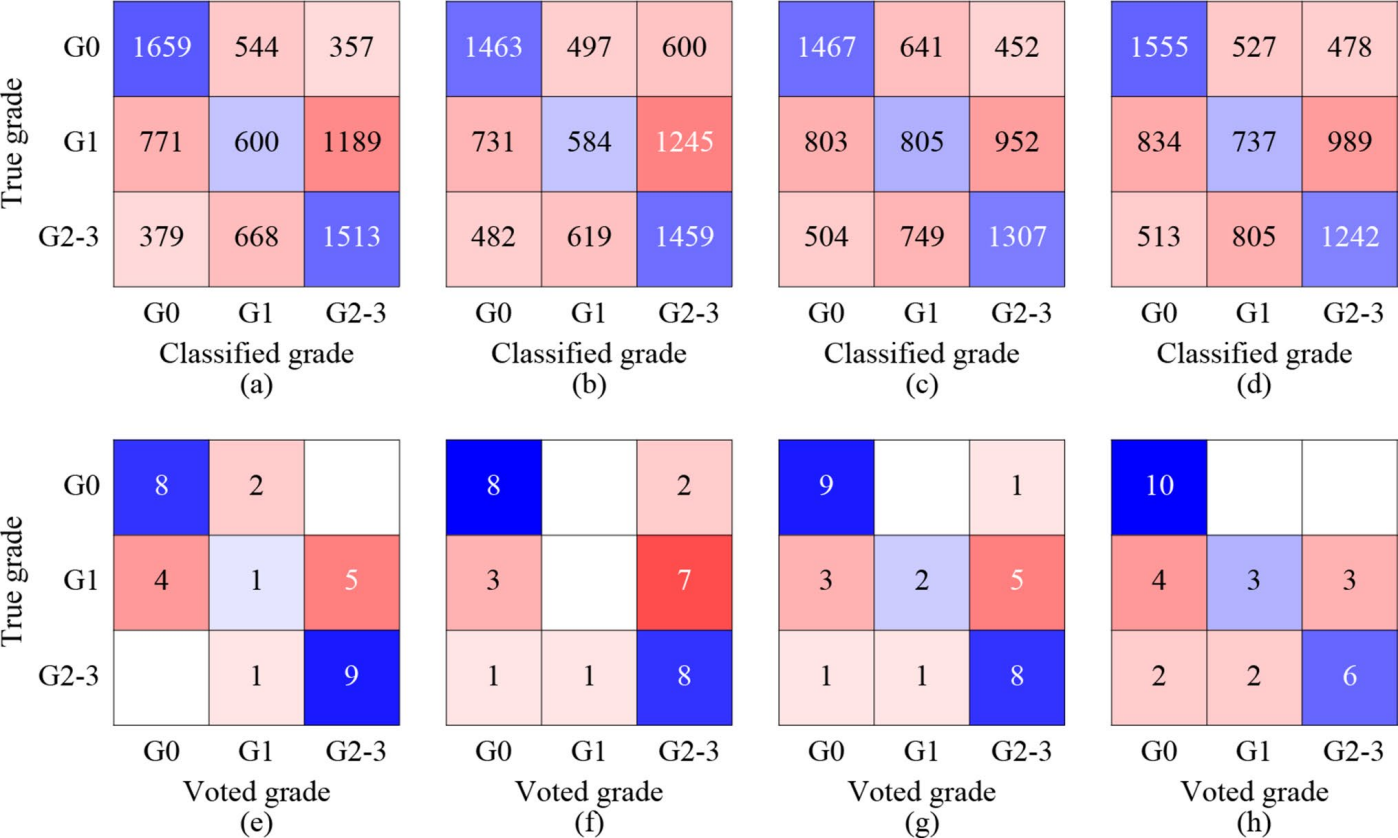
Classification results of the ROIs of **a** ultrasound images, **b** first-order moment maps, **c** fourth-order moment maps, **d** moment heatmaps obtained by the CNN, and those of cases obtained through a hard voting on the classified ROIs of **e** ultrasound images, **f** first-order moment maps, **g** fourth-order moment maps, and **h** moment heatmaps

Figure 5 shows the results of the fatty liver grade classification obtained using the CNN. In the CNN classification of the ROIs, the accuracies of the ultrasound images, first-order moment maps, fourth-order moment maps, and moment heat maps were 49.1%, 45.7%, 46.6%, and 46.0%, respectively. The ROIs of the ultrasound images and those of the first-order moment maps exhibited the highest and lowest classification accuracies, respectively. However, the difference between the input images appeared to be insignificant. In the hard voting for each case, the accuracies of the ultrasound images, first-order moment maps, fourth-order moment maps, and moment heat maps were 60.0%, 53.3%, 63.3%, and 63.3%, respectively. Hard voting significantly improved the classification accuracies of the fourth-order moment maps and moment heat maps.

**Fig. 6:**
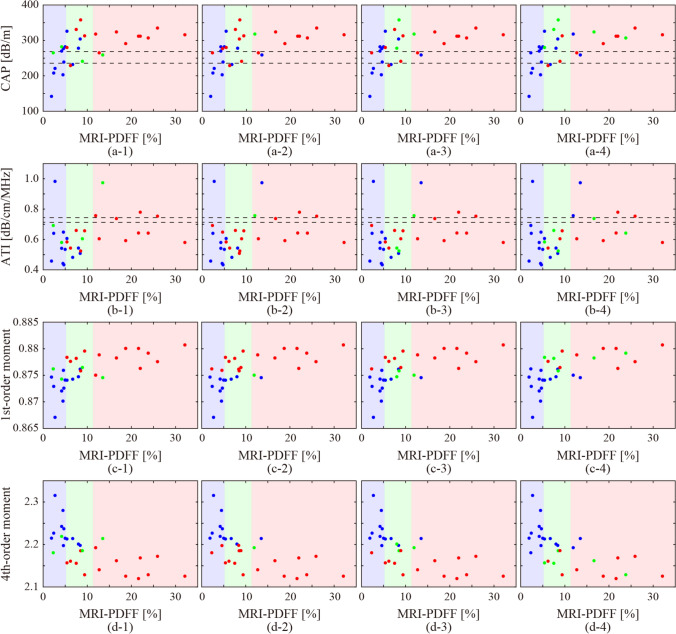
Relationship between the CNN classification of (1) colorized ultrasound images, (2) first-order moment maps, (3) fourth-order moment maps, and (4) moment heatmaps, with other indicators for liver steatosis, **a** CAP, **b** ATI, and the means of **c** first- and **d** fourth-order moments

To compare the CNN classification with the CAP and ATI, the colors of the plots in Fig. 3 were changed to blue, green, and red based on the grades of G0, G1, and G2-3 using the CNN, as shown in Fig. 6. The CNN classification results obtained using the ultrasound images exhibited a correlation with the CAP and the first- and fourth-order moments. When utilizing the first- and fourth-order moment maps, a correlation was observed with both the moments. However, in the case of the moment maps, the correlation between the results and liver steatosis indicators was poor.

In this study, CNN classification was conducted for each of the four types of input image. The differences in the classification results among the input image types were compared. In the case of colorized ultrasound images, there was a significant correlation between the classification results and CAP values, and mean moments, although only texture information was utilized for image classification. Regarding the cases classified as G1, the CAP values ranged from the cut-off values between G0 and G1 to those between G1 and G2-3, while the actual grades were G0 and G2-3. In the case of the first-order moment maps, the classification resulted in a binary classification of G0 and G2-3 based on the boundary value of the moment. The same trend was observed for the case of the fourth-order moment maps. Cases with mean moments close to the boundary values were classified as G1. Interestingly, the CAP values of the cases were greater than the cut-off values between G1 and G2-3. In the case of the moment heat maps, the classification results did not show a clear correlation with the CAP, ATI, or mean moments. The moment heat maps were classified based on the distribution and relationship of the first- and fourth-order moments, and not on the individual moments.

## Discussion

MRI-PDFF is considered the most reliable indicator in clinical practice for estimating hepatic fat deposition. Furthermore, a significant correlation has been observed between fat-droplet deposition and ultrasound attenuation. Therefore, a quantitative diagnosis based on CAP and ATI is also anticipated owing to the noninvasiveness and low cost of ultrasound scanners. However, the classification of fatty liver grades based on the cut-off values of CAP and ATI showed a low accuracy in this study. As shown in Fig. 3, the CAP and ATI values increased in accordance with the MRI-PDFF. Moreover, the means of the first- and fourth-order moments, which are indicators based on ES, increased and decreased, respectively. The correlation coefficients of the CAP, ATI, and means of the first- and fourth-order moments of each case with MRI-PDFF were 0.581, 0.250, 0.582, and − 0.515, respectively. The CAP and mean moments were significantly correlated with the MRI-PDFF. Therefore, if the effective cut-off values can be determined through future large-scale studies, the moments may also become effective indicators of liver steatosis.

In addition, the correlation coefficients of CAP, ATI, and the means of the first- and fourth-order moments of each case with elasticities measured with FibroScan were − 0.031, − 0.268, − 0.393, and 0.387, respectively. CAP and ATI were not correlated with liver stiffness. The correlation coefficients of the mean moments were higher than those of CAP and ATI, but sufficiently smaller when compared to the correlation coefficient with MRI-PDFF. Therefore, the ES such as moments may be more sensitive to steatosis than fibrosis.

This study focused on classification of parametric images into fatty liver grades using the CNN. While a qualitative correlation between both moments and fatty liver grades was observed, the quantitative aspect cannot be considered sufficient due to the limited number of cases. Therefore, the parametric images may be classified based solely on their moments, rather than being classified based on their fatty liver grades.

The first limitation of this study was the small amount of clinical data used. The number of cases were determined to be the same for each grade in a three-grade fatty liver classification to avoid learning bias due to imbalanced data. If the numbers of moderate and severe cases are sufficient, the proposed method can be evaluated using the actual four-grade classification.

The second limitation was the applicable liver conditions of the proposed method utilizing ES. The method focused on the increase in the first-order moment associated with the deposition of fat droplets. However, it is known that the first-order moment decreases as the replacement with fibrous tissues progresses. Therefore, the applicability of the proposed method is restricted to cases of mild fibrosis.

In the present study, the formation of moment maps and the extraction and selection of ROIs were automatically or randomly performed, while the only segmentation of liver regions was manually performed. Several segmentation algorithms of the liver region for ultrasound images have been developed [31, 32]. Therefore, the proposed fatty liver classification could be implemented in ultrasound scanners. As a result, it is anticipated that the ultrasound scanner will be able to automatically screen for liver steatosis during health checkups. The goal of this study was to contribute to the identification of individuals at risk and the early detection of MAFLD.

## Conclusions

In the present study, a method to classify ultrasound images into fatty liver grades based on the ES and CNN analyses was investigated. The texture of the ultrasound images of the liver varies owing to the deposition of fat droplets. Therefore, parametric images were formed based on ES of the ultrasound images to visualize the texture information affected by hepatic fat deposition. The first- and fourth-order moments, expected values of the exponential envelopes, and fourth power of the envelopes in the surrounding pixels were employed as the ES. Consequently, the first- and fourth-order moment maps and moment heat maps representing the relationships between the first- and fourth-order moments were formed as parametric images.

The ROIs were classified into fatty liver grades using the pretrained VGG-16. The classification accuracy of the ROIs for all types of parametric images was approximately 46%. The fatty liver grade for each case was determined by hard voting of the classified ROIs. The classification accuracy improved to approximately 63% when the fourth-order moment maps were used as the input images. For comparison with the ultrasound indicators used in actual clinical practice, the cases were also classified based on the CAP and ATI cut-off values. The classification accuracies were found to be 43.3% and 20%, respectively.

The moments of the ultrasound images served as indicators with a high correlation with the MRI-PDFF. Moreover, the moment maps and moment heat maps were highly effective as input images for the CNN classification of fatty liver grades.
